# Genome sequences of three genetic lineages of the fungus *Nothophaeocryptopus gaeumannii,* the causal agent of Swiss needle cast on Douglas-fir trees

**DOI:** 10.1128/mra.01008-23

**Published:** 2024-01-24

**Authors:** Nicolas Feau, Joey B. Tanney, Padmini Herath, Isabel Leal, Richard C. Hamelin

**Affiliations:** 1 Natural Resources Canada, Canadian Forest Service, Pacific Forestry Centre, Victoria, British Columbia, Canada; 2 Department of Forest and Conservation Sciences, Faculty of Forestry, University of British Columbia, Vancouver, British Columbia, Canada; 3 Département des Sciences du bois et de la Forêt, Faculté de Foresterie et Géographie, Université Laval, Québec, Canada; University of California Riverside, USA

**Keywords:** fungal pathogen, trees, Douglas-fir, ascomycete, endophytes

## Abstract

Here, we present the nearly complete genome sequences of the three main genetic lineages of *Nothophaeocryptopus gaeumannii*, an endophytic ascomycete fungus responsible for Swiss needle cast, a foliar disease that is emerging as a significant threat to the Douglas-fir tree in its natural distribution range.

## ANNOUNCEMENT

Many examples of geographically widespread, often genetically isolated, lineages have been described in fungi (1–3). For several decades now, an epidemic of Swiss needle cast (SNC) has been affecting Douglas-fir (*Pseudotsuga mensiezii*) in its native range in the Pacific Northwest region of Western North America (4). This disease is caused when the pseudothecia of the fungus *Nothophaeocryptopus gaeumannii* (Mycosphaerellaceae, Dothideomycetes, Ascomycota) physically obstruct the needle stomata, reducing its photosynthetic capacity (5). Two lineages of *N. gaeumannii* (lineage 1 c and 2) have been described on coastal Douglas-fir (*P. mensiezii* var. *mensiezii*); they have different levels of suitability to climates, increasing concerns that *N. gaeumannii* could rapidly adapt to environmental changes (6, 7). A third lineage, lineage 1i, was recently identified on the Rocky Mountain variety of Douglas-fir (*P. mensiezii* var. *glauca*) in the interior of British-Columbia (BC) and the Northern Rocky Mountains in the USA. In this study, we used Illumina PE and Oxford-Nanopore (ON) sequencing to generate a genome assembly for each of these lineages.

An axenic strain was obtained for each lineage by germinating on 1% water-agar media, an ascospore freshly released from a single pseudothecium on a 1-year-old Douglas-fir needle. The germinating ascospore was transferred on 2% malt extract agar amended with 0.03% yeast extract (ME). Fungal cultures were then identified at the lineage level using the real-time PCR assay described in reference (7). Flasks with 125 mL ME broth were then inoculated with subsequent strains and incubated at 20°C with 200 rpm shaking for 4 to 5 weeks. For Illumina sequencing, ~1.5 µg of genomic DNA was released using a cetyltrimethylammonium bromide chloroform protocol with isopropanol precipitation (8). DNA concentration and purity were measured with a NanoDrop ND-1000 spectrophotometer (Thermo Scientific). Paired-end (150 bp) libraries were prepared with the NEBNext Ultra II DNA Library Prep Kit for Illumina (New England Biolabs) and sequenced at the Génome Québec Innovation Center (Montréal, Canada) on an Illumina HiSeq X platform. DNA extraction and library preparation for Oxford Nanopore (ON) sequencing were performed at the Plateforme d’Analyses Génomiques of the Institut de Biologie Intégrative et des Systèmes (Université Laval, Quebec, Canada) as follows: high-molecular-weight DNA was isolated from ~30 mg of dried fungal mycelium using a chloroform and isopropanol procedure (9). DNA quality was assessed on a Femto Pulse instrument and a gDNA kit (Agilent) and resuspended DNA (~3 μg) and enriched for large fragments using Short Read Eliminator XS (SRE XS, PacBio). No shearing or size selection of the DNA was performed before library preparation. Multiplex libraries were constructed with 1 μg of DNA using the SQK-LSK109 and EXP-NBD104 kits from Oxford Nanopore Technologies. Sequencing was performed on an R9.4.1 flow cell with a GridION instrument (ON) and base calling was performed using Guppy ver. 6.4.2 set with the fast model.

For each lineage, ON reads were assembled into contigs with Flye v2.9.1 (10) with a genome size target of 35 Mb and default parameters. *De novo* assemblies were then polished with ON and Illumina reads using Racon version 1.4.13 (11) and Pilon (12), respectively. The size of the genome assemblies obtained in this study was slightly higher (35.5–35.6 Mb; Table 1) than what was obtained for the Illumina *de novo assembly* of the *N. gaeumannii* strain CBS 267.37 (34.0Mb; GenBank assembly GCA_002116385.1) (13). The high completeness of these genomes (Table 1) will enable comparative studies to identify factors related to the ecology and adaption of these lineages such as their capacity to tolerate different environmental conditions and ability to compete with other microorganisms during endophytic growth in conifer needles.

**TABLE 1:** Origin and general features of the genome sequences of Lineage 1c, 1i, and 2 of *Nothophaeocryptopus gaeumannii* isolated from Douglas-fir

	Lineage 1c	Lineage 1i	Lineage 2
Host	*P. mensiezii* var. *mensiezii*	*P. mensiezii* var. *glauca*	*P. mensiezii* var. *mensiezii*
Location	West Harrison lk., BC, Can. (N49.44, W121.85)	Enderby, BC, Can. (N50.56, W119.09)	Vancouver Island, BC, Can. (N49.34, W124.51)
DAOMC^*a*^ Identifier	252736	252735	252734
GenBank assembly	GCA_032718785.1	GCA_031771855.1	GCA_031771865.1
ON reads (SRA)	SRR26424609	SRR26369461	SRR26369462
No. of ON^*b*^ reads (N50 [bp])	137,939 (9,224)	91,752 (10,439)	239,558 (8,712)
Illumina reads (SRA)	SRR26424608	SRR26363295	SRR26363294
No. of Illumina reads	8,016,282	8,980,234	7,094,830
Assembly size (Mb)	35.47	35.59	35.51
Coverage	20.0X	14.0X	46.0X
No. of contigs	50	61	42
N50 (Mb)	1.67	1.84	2.70
L50	7	7	6
Length longest contig (Mb)	6.23	4.10	3.67
No. of contigs > 1 Mb	17 (34.0%)	11 (18.03%)	15 (35.71%)
GC content	52.52%	52.52%	52.52%
% BUSCO (C,D,F,M)^*c*^	99.87% (755,2,2,1)	99.74% (753,3,3,2)	99.74% (755,2,1,2)

^*a*^ Canadian Collection of Fungal Cultures, https://agriculture.canada.ca/en/science/agriculture-and-agri-food-research-centres/canadian-collection-fungal-cultures-daomc.

^*b*^ ON, Oxford Nanopore.

^*c*^ Benchmarking Universal Single-Copy Orthologs (BUSCO) completeness evaluated with a set of 758 gene orthologs in fungi C, complete; D, duplicated; F, Fragmented; M, Missing.

## Data Availability

All the data are available under BioProject number PRJNA971752; The individual SRA and GenBank assembly numbers are included in Table 1. The script for the assembly pipeline is available in the GitHub repository https://github.com/feaunico/SNC_denovo_assemblies.
